# Imagery Ability and Imagery Perspective Preference: A Study of Their Relationship and Age- and Gender-Related Changes

**DOI:** 10.1155/2019/7536957

**Published:** 2019-07-31

**Authors:** Karen P. Y. Liu, Monica Lai, Shirley S. M. Fong, Michelle Bissett

**Affiliations:** ^1^School of Science and Health, Western Sydney University, Australia; ^2^Translation Health Research Institute, Western Sydney University, Australia; ^3^School of Nursing and Healthcare, Hong Kong Nang Yan College of Higher Education, Hong Kong; ^4^School of Allied Health Science, Griffith University, Australia

## Abstract

This study examined if imagery ability (i.e., vividness and temporal congruence between imagined and executed knee extensions) and imagery perspective preference were affected by ageing and gender. Ninety-four participants, 31 young, 43 intermediate, and 20 older adults completed the Vividness of Movement Imagery Questionnaire-2 and a knee extension temporal congruence test to reflect on their imagery ability and an imagery perspective preference test. Male participants had a better imagery ability than the female participants (*F* (4, 85) = 2.84, *p* = .029, *η*^2^ = .118). However, significant age-related changes in imagery ability were not found in the three age groups. Change in imagery perspective preference with a trend towards an external imagery perspective was observed with ageing (*F* (3, 89) = 3.16, *p* = .028, *η*^2^ = .096) but not between male and female. The results suggest that imagery ability may be preserved with ageing. As individuals age, their preference for using an imagery perspective shifts from a more internal to a more external perspective. This understanding is important when designing future imagery research and real-life application or clinical intervention.

## 1. Introduction

In the past five decades, mental imagery has gathered a large body of research supporting its use in facilitating performance of motor and cognitive tasks (for meta-analytical reviews, see [1, 2]). Motor imagery, which involves simulating an action without its physical execution [3], has been of particular interest to researchers in fields of sport and exercise and clinical science due to its increasingly demonstrated efficacy in enhancing athletic performance (e.g., [4]) and motor performances of clients with a range of functional problems (e.g., [5–10]).

On account of the growing body of research, there is now an increased understanding of what factors modulate imagery ability to enhance motor performance. These factors include the imagery perspective used [11] and the imagery ability of the individual [12]. Imagery perspective refers to whether an image is generated as an internal view (i.e., first-person perspective) or an external view (i.e., third-person perspective). Imagery ability is commonly referred to as the mental chronometry and vividness of the image. Mental chronometry is regarded as the time course of mental operations between simulated and real movements and is measured by a relatively objective temporal congruence test [13]. The temporal congruence test records the duration of physically executed and imagined movements, and it compares between real and imagined movement times. Vividness refers to the image clarity or perceived sensation intensity [3] and is measured by subjective questionnaires such as the Vividness of Movement Imagery Questionnaire-2 [14], which assesses vividness of the image when using an internal and external visual perspective (external visual imagery (EVI) and internal visual imagery (IVI)) and when using kinesthetic imagery (KI) (i.e., feeling the movement).

Previous studies have demonstrated that ageing causes a slight decline in imagery ability. For example, the vividness of imagery, measured in studies by Malouin, Richards, and Durand [15] and Mulder, Hochstenbach, Van Heuvelen, and Den Otter [16], was found to be altered with ageing. The former study showed a change in imagery quality, and the latter showed a significant decline in using an internal perspective. Researchers are also keen to look at the differences in male and female, and some studies reported that the results changed as different types of assessments were used [17].

Schott [18] found imagery ability, as indicated by temporal congruence between imagined and real motor tasks and vividness of the image, was negatively impacted in adults aged 70 years and over. Furthermore, Kalicinski, Kempe, and Bock [19] found an age-related decline in the vividness of the image. However, temporal congruence in simple and everyday-like tasks was less affected.

Despite the increased knowledge, the age-related changes of imagery perspective preference and the role of imagery perspective preference in imagery ability are largely unknown. Although it has been proposed that preference for using an internal or external imagery perspective is identifiable through the ability to use these perspectives [20], Callow and Roberts [21] found only a moderate correlation between the two, hence indicating that internal or external imagery perspective may have different constructs. Moreover, the study only examined young athletes with a mean age of 19.60. To our knowledge, there is no report examining how ageing and sex might affect the imagery perspective preference. The understanding of the role of imagery perspective preference, which may prove important when planning imagery research and intervention, on imagery ability is also minimal. Therefore, the objectives of this study are to investigate age-related changes in imagery ability (as measured by mental chronometry and vividness) and imagery perspective preference and to examine the relationship of preference for a visual imagery perspective with mental chronometry and vividness. It also explores the differences between male and female.

## 2. Methods

### 2.1. Participants

Ninety-four volunteers participated in the study: 31 young (22 females; age range = 18 − 35 years; mean age = 26.52 ± 5.8 years); 43 intermediately aged (29 females; age range = 36 − 60 years; mean age = 46.37 ± 8.6 years), and 20 old (9 females; age range ≥ 60 years; mean age = 69.55 ± 7.0 years). They were recruited by posting flyers in the Western Sydney University campuses. Using a snowball sampling method, participants referred their friends and family for the study. All participants were healthy through self-report, had no neurological conditions and musculoskeletal limitations, and showed normal cognitive function as screened by attention (mean score = 7.70 ± .710; with cutoff of 6), comprehension (mean score = 5.97 ± .17, with cutoff of 5), and short-term memory (mean score = 11.69 ± .74, with cutoff of 10) items of the Cognistat [22, 23]. No statistical significance was found in the cognitive function of the three age groups and the two gender groups, except for comprehension (Table 1). The post hoc Tukey HSD test showed that significant differences occurred between the young group and the intermediately aged group (*p* = .040) and between the young and old groups (*p* = .049). No participant reported recent knee surgery or knee extension dysfunction which might have impacted on their ability to perform knee extensions for mental chronometry testing. All participants provided informed written consent, and the study protocol was approved by the local ethics committee.

### 2.2. Measures

The imagery ability was measured in terms of the individual's imagery of a real movement (the mental chronometry) and a self-reported vividness of imagery using a questionnaire (Vividness of Movement Imagery Questionnaire-2). The participants' preference over external or internal imagery is also measured.

#### 2.2.1. Imagery Ability: Mental Chronometry

This assessment provides explicit information on the temporal coupling between real and simulated movements, and it is a widely used measure for assessing imagery [13]. Measurement involves two chronometric tests: (1) the Time-Dependent Motor Imagery screening test which quickly determines whether or not the individual understands the instruction and can perform motor imagery and (2) the temporal congruence test which compares real and simulated movement times. The protocol developed by Malouin et al. [13] was used in this study. Both chronometric tests have provided reliable measures for healthy participants and participants poststroke [13].

In the current study, mental chronometry measurement of knee extensions (dominant side) was selected for its ease of testing amongst all age groups. To identify the dominant leg for measurement, the Foot Preference Questionnaire [24] was used before testing. Mental chronometry was then measured in a similar procedure used by Malouin et al. [13]. The Time-Dependent Motor Imagery screening test was administered first, and if the participant increased the number of imagined knee extensions with the increasing time periods, the temporal congruence test, which compares real and simulated movement times, was administered.

During testing, participants sat on a chair with a backrest. In the Time-Dependent Motor Imagery screening test, participants were instructed to imagine knee extensions (dominant side) over time periods of 15, 25, and 45 seconds, presented randomly to avoid time duration predictions. During testing, participants closed their eyes during imagery of knee extensions and signaled either verbally (such as counting) or physically (such as nodding head) each time their knee was extended mentally. The temporal congruence test involved participants imagining and then physically executing two series of five knee extensions. A minimum of 30-second rest intervals between each series and each imagined and executed condition was provided. As with the Time-Dependent Motor Imagery screening test, participants signaled when imagining a knee extension.

The mean of the two series of imagined and executed conditions was computed. The temporal congruence between the imagined and executed knee extensions was represented by the calculated imagined/executed ratios, with a score of 1 representing perfect temporal equivalence between imagined and executed movements.

#### 2.2.2. Imagery Ability: Vividness of Movement Imagery Questionnaire-2

Each participant's vividness of imagery was measured using the Vividness of Movement Imagery Questionnaire (VMIQ-2) [14]. The VMIQ-2 assesses vividness while using EVI, IVI, and KI in motor tasks such as walking, running, kicking a stone, and bending to pick up a coin. A five-point scale (1 = perfectly clear and vivid; 5 = no image at all) was used by the participants to rate their vividness in EVI, IVI, and KI for each task. A lower score represents clearer vividness of imagery.

#### 2.2.3. Imagery Perspective Preference

To determine preference for using either an internal or external imagery perspective, all participants rated their perspective preferences by visually imagining the 12 items in the VMIQ-2 [21]. Preferences were anchored at 0 (strong internal visual preference), 3 (moderate internal visual preference), 5 (no preference), 7 (moderate external visual preference), and 10 (strong external visual preference). In determining a difference in imagery perspective preference amongst the three age groups, we used the total scores of the visual imagery perspective preference scale, which were a minimum of 0 and a maximum of 120. A total score of 60 represents no preference. A total score less than 60 represents an internal preference and greater than 60 represents an external preference.

### 2.3. Procedure

After obtaining informed consent, all participants who fulfilled the selection criteria were asked to provide their age, sex, occupational status, and highest education level. Measurement sessions were administered by trained clinicians either at participant homes, community centers, or university campus rooms. Participants were asked to complete the questionnaires without conferring with others, and each participant was tested individually for the knee extension mental chronometry test. To prevent ordering effects, the testing procedure was counterbalanced by administering either the VMIQ-2 or the mental chronometry test first. A randomized sequence of the assessments was used.

### 2.4. Data Analyses

Descriptive statistics were reported for all measures across the three age groups and gender. Imagery ability and imagery perspective preference, being in two different constructs, were analyzed separately. The normality of the measures of the three age groups was established using the Shapiro-Wilk tests.

As there was a significant difference in comprehension scores of the Cognistat in the three age groups, the score was put as a covariate when comparing the results of the three age groups. Therefore, the multivariate analysis of covariance (MANCOVA) test was used to review if there was any difference in the imagery ability as measured by mental chronometry (the temporal congruence ratio) and VMIQ-2 (the imagery vividness for EVI, IVI, and KI) across the age groups and the analysis of covariance (ANCOVA) test was used to test if there was any difference in the imagery perspective preference as measured by the VMIQ-2 across the age groups. The multivariate analysis of variance (MANOVA) test and the analysis of variance (ANOVA) test were used to test if there was any difference in the imagery ability and the imagery perspective preference in the two gender groups.

Partial eta squared (*η*^2^) indicating the effect size for those with significant differences was reported. The post hoc Tukey HSD test was then adopted to show the differences in imagery ability and imagery perspective preference in any two age groups.

Pearson product-moment correlations were used to show the relationship of imagery ability and imagery perspective preference. The statistical significance level was set at *p* < .05.

## 3. Results

Across all age groups, the temporal congruence ratio measured by the mental chronometry test indicating the imagery ability was 1.21 ± .37, indicating a longer imagined time than executed time. The older age group had the best temporal congruence while the young age group had the lowest temporal congruence (Table 2). For the results of imagery vividness as measured by the VMIQ-2, the mean score for EVI was 27.19 ± 12.49, for IVI was 23.61 ± 11.49, and for KI was 29.59 ± 13.73 in all age groups. By inspecting the obtained means, the young age group had the best imagery vividness (Table 2). The older age group had the lowest imagery vividness for IVI and KI, while the intermediate age group had the lowest imagery vividness for EVI. However, with the covariate of the comprehension score, the results of MANCOVA did not reveal any significant difference in the imagery ability amongst the age groups (*F* (8,170) = 1.25, *p* = .27).

Male participants had a better imagery ability in both temporal congruence as measured by the mental chronometry and imagery vividness for EVI, IVI, and KI measured by the VMIQ-2 than the female participants (Table 2). The results of the MANOVA test revealed an overall significant difference between male and female (*F* (4, 85) = 2.84, *p* = .029, *η*^2^ = .118). It was further revealed that significant differences were found in the temporal congruence (*p* = .031, *η*^2^ = .076), but not in imagery vividness for EVI, IVI, and KI.

The obtained mean in imagery perspective preference across all groups was 48.86 ± 30.79. The average score on each item was approximately four which lies between the “moderate internal visual preference” and “no preference” anchors on the scale. With the increasing age ranges, the mean scores became higher (Table 2), indicating an increasing preference for EVI with ageing. ANCOVA revealed significant difference amongst the age groups (*F* (3, 89) = 3.16, *p* = .028, *η*^2^ = .096). The post hoc Tukey HSD tests revealed that the difference in imagery perspective preference was significant between the young and older age groups (*p* = .010) and between the intermediate and older age groups (*p* = .048).

Female participants seemed to indicate an increasing preference for EVI than male participants although the result of ANOVA did not reveal any significant difference (*F* (1, 92) = .12, *p* = .733).

No significant correlations were found between imagery perspective preference scores and temporal congruence and between imagery perspective preference scores and imagery vividness for KI. However, significant moderate correlations were found between imagery perspective preference and imagery vividness for EVI (*r* = ‐.35, *p* = .001) and between imagery perspective preference and vividness for IVI (*r* = .33, *p* = .001).

## 4. Discussion

The study did not find age-related changes in imagery ability although there was a trend showing that the older age group had a better temporal congruence while the young age group had a better imagery vividness. However, preference for an imagery perspective was found to change with ageing. A significant trend was observed in preference moving towards an external imagery perspective with ageing. When compared between male and female, the male participants had significantly better imagery ability, particularly in temporal congruence. No significant differences were found in imagery perspective preference between the two groups although the female participants showed a trend in preference moving towards an external imagery perspective. In regard to the relationship of imagery perspective preference and imagery ability, a significant moderate correlation was found between imagery perspective preference and imagery vividness in internal and external visual imagery. A preference towards external imagery perspective was significantly correlated with imagery vividness in external visual imagery. A similar trend of a significant correlation was found for those with a preference towards internal imagery perspective; they had a better score in the imagery vividness in internal visual imagery.

The results of the present study for age-related imagery changes were somewhat conflicting to previous studies. For temporal congruence, the data revealed that as participants aged, their timing for imagining knee extensions became closer to their timing for executing knee extensions although the results were not significant. In contrast, previous researches show a greater difference between imagined and executed movements in the oldest age groups [18, 25, 26]. A likely reason for such discrepancy may be the relative ease of the knee extension task in the current study. As Saimpont et al. [3] concluded, older adults seem to have a preserved ability in the mental chronometry for simple movements, but an altered ability in constrained and unusual movements. Furthermore, the complexity and duration of the motor task are also said to influence mental chronometry [19, 27]. Therefore, it appears that in the present study, the knee extension task's simplicity and short duration enabled a high temporal equivalence for the older participants. The question remains, however, as to why the older adults showed a trend with better mental chronometry results. Possibly, the more years of experience acquired by the older adults had helped increase their temporal equivalence as stated by Guillot, Louis, and Collet [28]. This information is beneficial for clinical practitioners, as it supports gathering an appropriate history of task experience from clients and designing imagery tasks appropriate for the older age group. The results are also supportive of helping older adults relearn motor tasks in simple and short steps, in which older participants showed high temporal equivalence.

No significant difference was found for imagery vividness amongst the three age groups. However, there was a trend that the older participants had a lower imagery vividness for internal visual imagery and kinesthetic imagery. This was both in accordance and in contrast with the findings of Mulder et al. [16], where no difference was found in vividness using an external perspective, but an age-related shift was found from better internal visual imagery vividness to better external visual imagery vividness. Mulder et al. [16] discussed that a possible reason for this shift might be due to a decrease in physical activity (commonly observed with ageing) having a negative influence on the ability to imagine self-movement. It was suggested that nonphysically active older persons might spend more time watching others move around and hence may be better in using an external perspective than an internal perspective [16]. Indeed, this may point to the reason for the trend as shown by the raw data of the current study although we did not collect the information on their physical activity level to verify this hypothesis. Nevertheless, unlike the population of elderly described by Mulder et al. [16], most participants in the older age group of the present study were currently physically active with the mean age of only 69.55. Therefore, it is likely that the maintained physical activity and comparatively younger age may have helped sustained vividness using an internal perspective.

Surprisingly, despite the sustained internal visual imagery ability, the older age group showed a significant preference for using an external visual perspective in comparison to the internal visual perspective preferred by the young age group. This may be of interest to clinical practitioners implementing imagery in therapy, as older clients with motor impairments may more readily use an external imagery perspective. This perspective was found to be more effective for learning complex and form-based tasks, which is important for clients after stroke, as daily functional movements may be deemed as complex [29]. Nilsen et al. [10], however, found no difference in efficacy using either perspective for people after stroke. Nevertheless, this understanding of a preferred external imagery perspective in the elderly may help to guide future imagery scripts that are tailored to generic preferences.

Also of interest for imagery scripts was that the younger participants' preference for an internal perspective was only moderate. A previous study has found certain imagery perspectives to be beneficial for certain tasks [11]. Therefore, implementing either internal imagery or external imagery by its efficacy for a certain task could more likely result in compliance of young adults, as they may not have a strong preference for an internal imagery perspective. Young participants or an individual who is learning complex motor skills might found the internal or external imagery useful in learning the skills [30]. Young participants in the current study indeed did not show a strong preference, despite the previous speculation that IVI may be a default perspective (Morris & Spittle, 2001, as cited in [31]). This speculation was supported by findings from Spittle and Morris [31] that young adults used more internal imagery when imagining sports skills. However, imagery perspective preference was measured differently in the current study, with clear instructions on participants indicating their “preference” on the imagery perspective preference scale, as opposed to Spittle and Morris [31] where participants were provided with concurrent verbalization during their imagery, and the imagery perspective was then verified by a rating scale and retrospective verbalization of imagery perspective use completed after the task. Additionally, the present study measured the visual imagery perspective preference, while the previous study did not specify the imagery modality (i.e., visual or kinesthetic). Future research investigating imagery perspective preference measurement may provide an understanding of what is a more accurate measure.

This study revealed a difference in imagery vividness but not imagery perspective preference between male and female participants. Male was found to have better imagery vividness in this study. This result is not consistent with the previous studies [32, 33] using a questionnaire-based assessment to test imagery ability and found that female had better imagery ability. As indicated by Campos [32], male participants showed better imagery ability if a performance-based type of assessment was used. In our study, we used a performance-based mental chronometric test to assess imagery vividness. The performance-based imagery test could be a more accurate assessment to reflect on a person's imagery ability.

Assessing the imagery perspective preference is important. The objective was to investigate if imagery perspective preference was related to imagery ability. While no relationship was found with temporal congruence, a moderate relationship was found with vividness for external and internal visual imagery. The results showed a significant negative moderate correlation between imagery perspective preference and vividness for external visual imagery and a significant positive moderate correlation between imagery perspective preference and vividness for internal visual imagery. The moderate correlations support the findings and suggestion made by Callow and Roberts [21]. Although a relationship exists, imagery perspective preference and imagery ability (regarding vividness) may be of different constructs. Therefore, it is recommended that imagery perspective preference is measured separately to imagery ability if researchers and practitioners wish to identify perspective preferences.

For temporal congruence as measured by the mental chronometry, a lack of relationship with imagery perspective preference may not be surprising, particularly, since the temporal congruence between imagined and executed movements using both an internal and external perspective was not examined. While mental chronometry using both perspectives has been previously studied [34], it will be helpful in the future study to also examine if a higher preference in using an imagery perspective would be related to a more temporal equivalence when using that perspective.

The results were limited by only measuring vividness and temporal congruence as determinants of imagery ability. In order to capture a more comprehensive view of imagery ability, it would have been beneficial to use a measure of controllability which is also regarded as a component of imagery ability [35] and an accuracy measure which is objective, requiring the individual to simulate spatial manipulation of a visual stimulus.

Some caution must also be taken concerning the generalizability of the present findings. The arbitrary age cutoff of the three age groups may not be the most appropriate and cannot represent truly people of the particular age group. There was an imbalance of male and female participants especially in the young group. As previously discussed, the sample from the older age group was mostly physically active and, therefore, may not be a true representative of the older people. Moreover, participants were recruited by convenience sampling. Therefore, the representation of the results to a wider population may be limited.

## 5. Conclusions

In light of the findings in the present study, imagery ability seems to be preserved with ageing. Male seems to have a better imagery ability than female. For imagery perspective preference, a significant age-related change was observed, with results indicating that as individuals age, their preference for using a visual imagery perspective shifts from a more internal perspective to a more external perspective. This understanding is important when designing future imagery research and real-life application or clinical intervention, as the consideration of preferences may show more efficacious results. Also of interest is that the present study's findings suggest that across young, intermediate, and older ages, preference for using an internal or external imagery perspective is moderately related to better imagery vividness using that perspective.

## Figures and Tables

**Table 1:** Results of Cognistat.

Measures	Age				Gender		
	<35	36-60	>60	*p* values (differences in age groups)	Female	Male	*p* values (differences between genders)
	M ± SD	M ± SD	M ± SD		M ± SD (95% CI)	M ± SD (95% CI)	
Attention	7.90 (.40)	7.67 (.75)	7.45 (.89)	.081	7.65 (.77)	7.73 (.67)	.592
Comprehension	6.00 (.00)	6.00 (.00)	5.85 (.37)	.003^∗^	5.94 (.24)	5.98 (.13)	.276
Memory	11.80 (.55)	11.72 (.77)	11.45 (.89)	.241	11.65 (.85)	11.71 (.67)	.685

∗ denotes *p* < .05.

**Table 2:** Results.

	Measures	Age				Gender		
		<35	36-60	>60	*p* values (differences in age groups)	Female	Male	*p* values (differences between genders)
		M ± SD (95% CI)	M ± SD (95% CI)	M ± SD (95% CI)		M ± SD (95% CI)	M ± SD (95% CI)	
Mental chronometry	Temporal congruence	1.32 ± .45 (1.16, 1.49)	1.17 ± .28 (1.09, 1.26)	1.11 ± .37 (.93, 1.29)	.142	1.28 ± .41 (1.18, 1.39)	1.09 ± .24 (1.00, 1.17)	.012^∗^

VMIQ-2	Vividness for external visual imagery	24.42 ± 10.17 (20.69, 28.15)	29.26 ± 12.61 (25.38, 33.14)	27.05 ± 15.04 (20.01, 34.09)	.429	27.98 ± 12.29 (25.16, 31.63)	25.79 ± 12.88 (21.30, 30.29)	.338
	Vividness for internal visual imagery	21.81 ± 10.41 (17.99, 25.63)	23.47 ± 10.90 (20.11, 26.82)	26.70 ± 13.86 (20.21, 33.19)	.525	25.43 ± 12.12 (22.29, 28.71)	20.38 ± 9.49 (17.07, 23.69)	.039^∗^
	Vividness for kinesthetic imagery	27.29 ± 13.52 (22.33, 32.25)	30.56 ± 13.74 (26.33, 34.79)	31.05 ± 14.26 (24.38, 37.72)	.690	31.30 ± 14.92 (27.83, 35.69)	26.56 ± 10.88 (22.76, 30.36)	.080
	Imagery perspective preference	40.81 ± 32.91 (28.73, 52.88)	46.84 ± 28.56 (38.05, 55.63)	65.70 ± 26.71 (53.20, 78.20)	.028^∗^	49.68 ± 29.80 (41.23, 56.74)	47.41 ± 32.87 (35.94, 58.88)	.285

VMIQ-2: Vividness of Movement Imagery Questionnaire-2. ∗ denotes *p* < .05.

## Data Availability

The data used to support the findings of this study are included within the article. Requests for access to these data can be made to Karen Liu, karen.liu@westernsydney.edu.au.
